# Supplementary material for: Birth season shapes the infant metabolome and development in Tanzania: a secondary explorative analysis of the early life interventions for childhood growth and development in Tanzania (ELICIT) trial
Source: Nat Commun. 2025 Dec 13;16:11469. doi: 10.1038/s41467-025-66268-9 (PMC12749142; doi:10.1038/s41467-025-66268-9)

Birth season shapes the infant metabolome and development in Tanzania: a secondary explorative analysis of The Early Life Interventions for Childhood growth and development in Tanzania (ELICIT) trial

Supplementary Figures

Fig.S1.

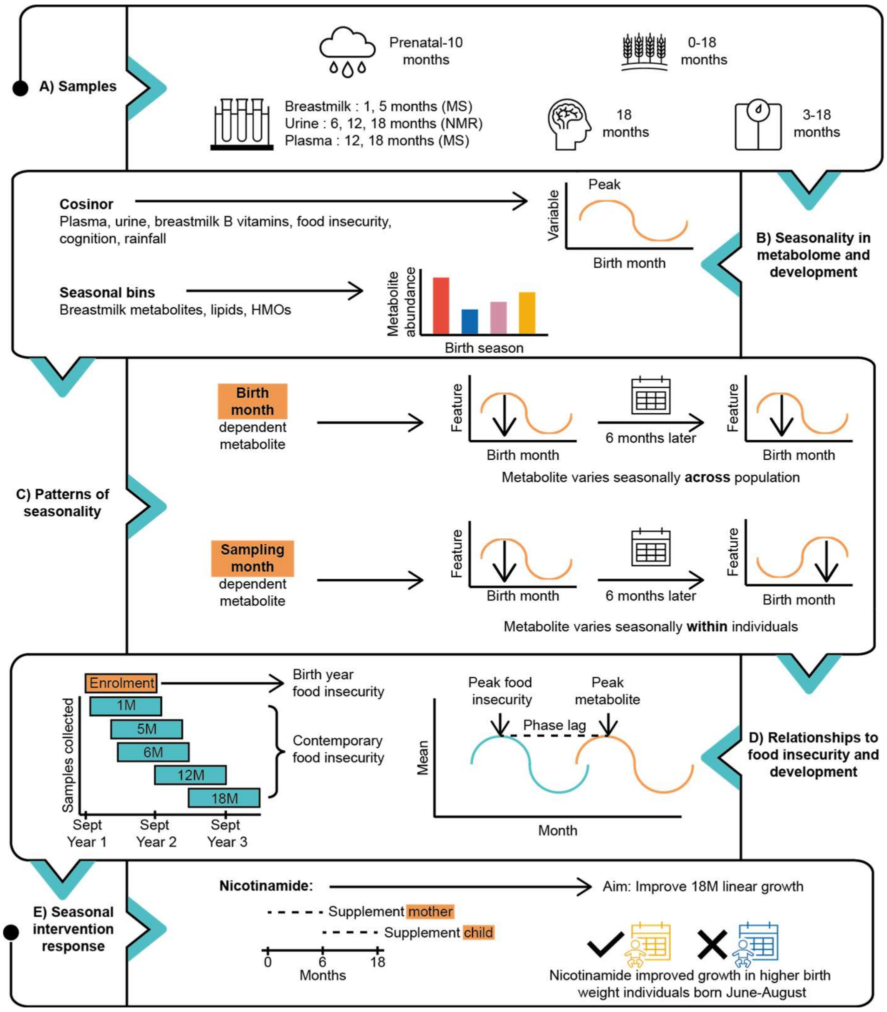

Schematic of manuscript analysis. A) Details of sample collection including Haydom, Tanzania rainfall data from 2016-2018, monthly maternal food insecurity reports across an 18-month period from birth, biological samples (breastmilk at one and five months post-partum with metabolomes measured by LC-MS and FIA-MS; urine at six, 12, and 18 months measured by ^{1}H NMR; plasma at 12 and 18 months measured by LC-MS and FIA-MS), cognitive outcomes at 18 months, and anthropometry (LAZ, WAZ, MAZ, HCZ) measured every three months from three to 18 months. **B**) Cosinor analysis used to determine 12-month variation by birth month or Kruskal-Wallis comparison of seasonal bins for variables sampled in small subset. **C**) Seasonal patterns determined as birth-month dependent if pattern remains static across sampling times; determined as sampling-month dependent if peak birth month shifts ~six month between sampling times. **D**) Cross correlation analysis assessed time-shifted relationships between metabolites and food insecurity (over a 12-month window). Phase lag represents the time difference between the peak birth month of metabolite and month of peak food insecurity. **E**) Nicotinamide intervention aiming to improve 18-month linear growth. Given as 250 mg daily tablets for six months post-delivery while breastfeeding, followed by direct infant

supplementation (100 mg sachets) from six to 18 months. Improved growth observed in subset of participants.

Fig.S2.

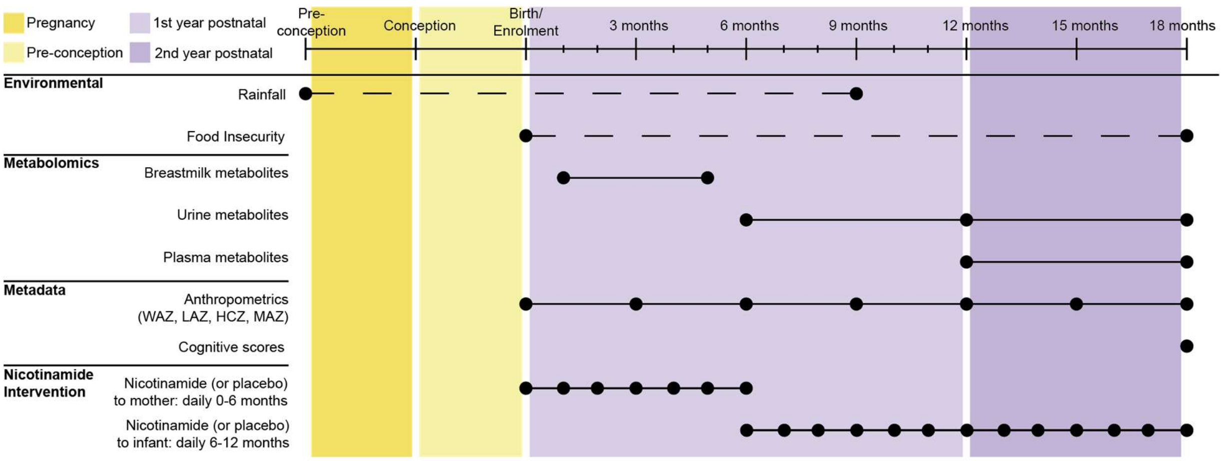

ELICIT study design and overview. Mother-infant dyads were enrolled within 14 days of birth beginning with the enrolment period lasting from September 2017-August 2018. Dots indicate time point of data collection. Colored by period of early life.

Fig.S3.

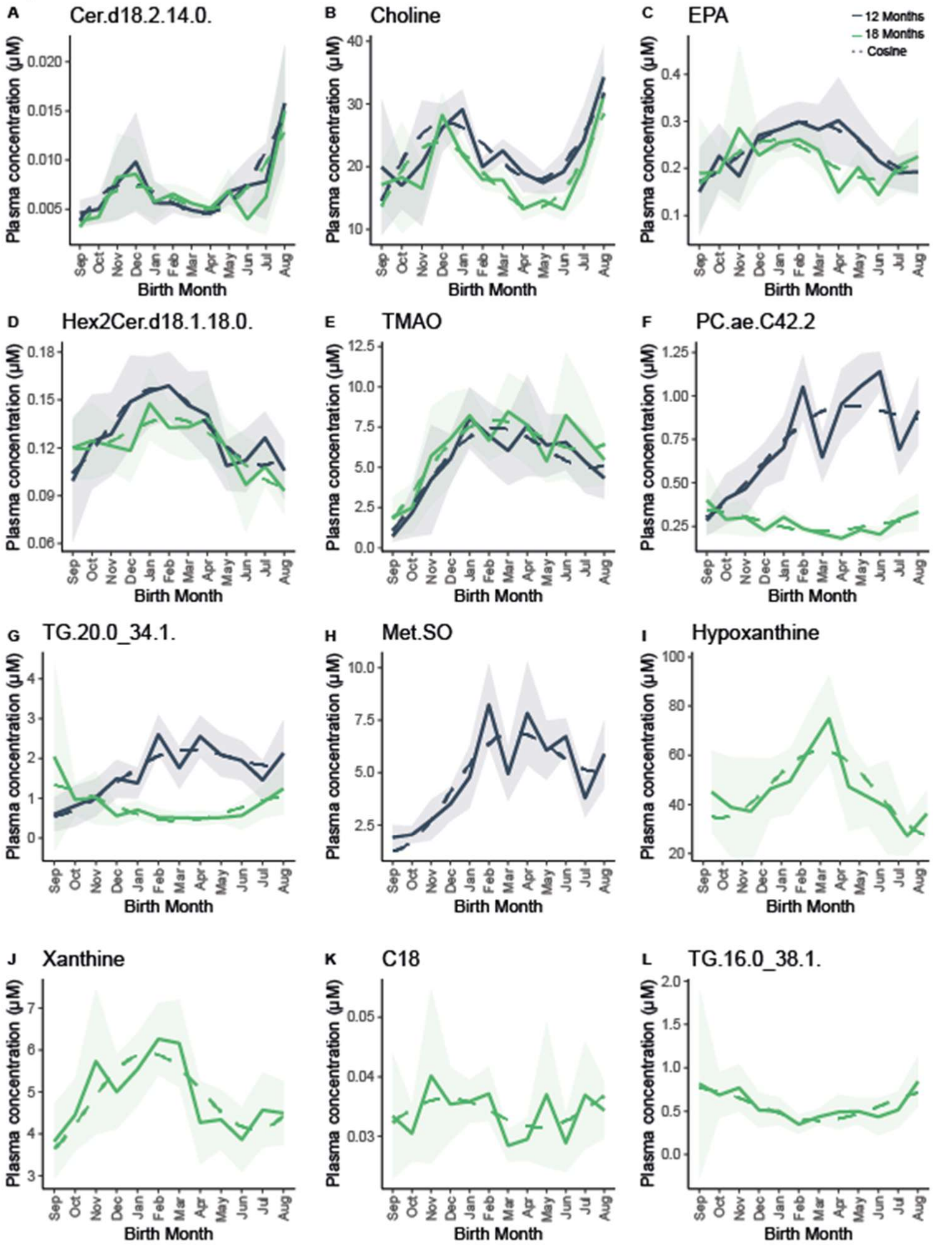

Variation in plasma metabolome at 12 and 18 months is influenced by the month of birth
A-L) Mean plasma metabolite concentrations by birth month at 12 (grey) and 18 (green) months (bold line; n = 199) following cosine distribution (dashed line) with 95% CI overlaid (shaded). Metabolites plotted at time point observed to follow a seasonal waveform based upon month of birth, from cosinor analysis (plasma n = 199; $p < 0.05$ & $q < 0.15$ following Benjamini-Hochberg correction at ≥ one sampling point by extra-sums-of-squares F-test; exact p-values are provided in Supplementary Table 2).

Fig.S4.

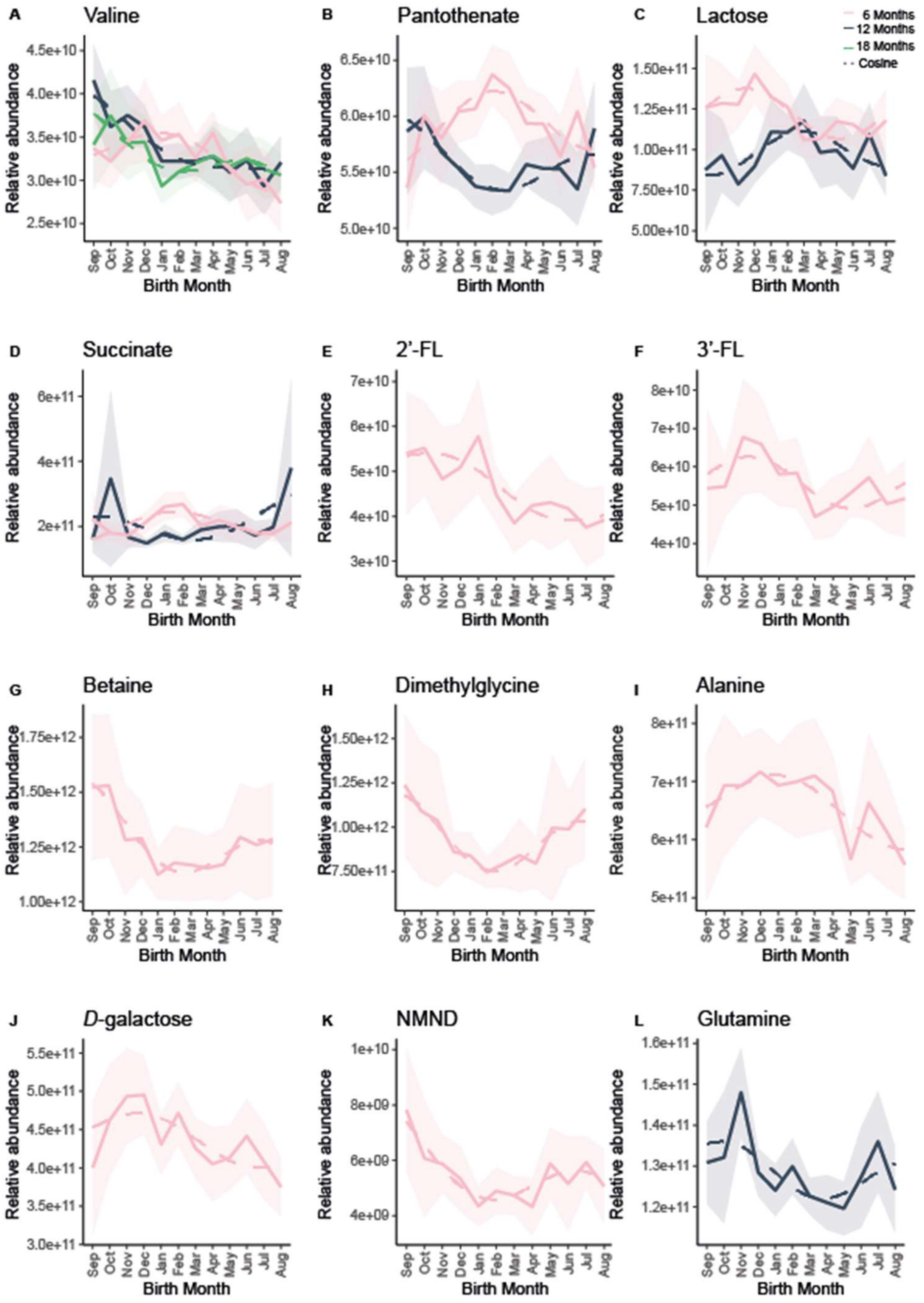

A
Valine
B
Pantothenate
C
Lactose
6 Months
12 Months
18 Months
Cosine
D
Succinate
E
2'-FL
F
3'-FL
G
Betaine
H
Dimethylglycine
I
Alanine
J
D-galactose
K
NMND
L
Glutamine
Relative abundance
Birth Month
Sep
Oct
Nov
Dec
Jan
Feb
Mar
Apr
May
Jun
Jul
Aug

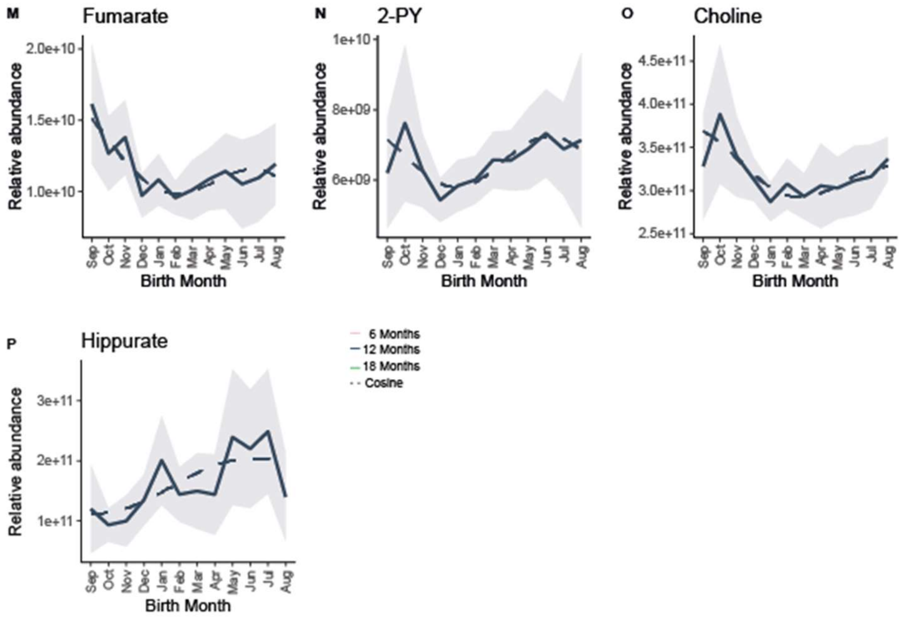

Variation in urinary metabolome at 6, 12 and 18 months is influenced by the month of birth

A-P) Mean urinary metabolite relative abundances by birth month at six (pink), 12 (grey) and 18 (green) months (bold line) following cosine distribution (dashed line) with 95% CI overlaid (shaded). Metabolites plotted at time point observed to follow a seasonal waveform based upon month of birth, from cosinor analysis (6-month n = 278; 12-month n = 270; 18-month n = 266; $p < 0.05$ & $q < 0.15$ following Benjamini-Hochberg correction at ≥ one sampling point by extra-sums-of-squares F-test; exact p-values are provided in Supplementary Table 3).

Fig.S5

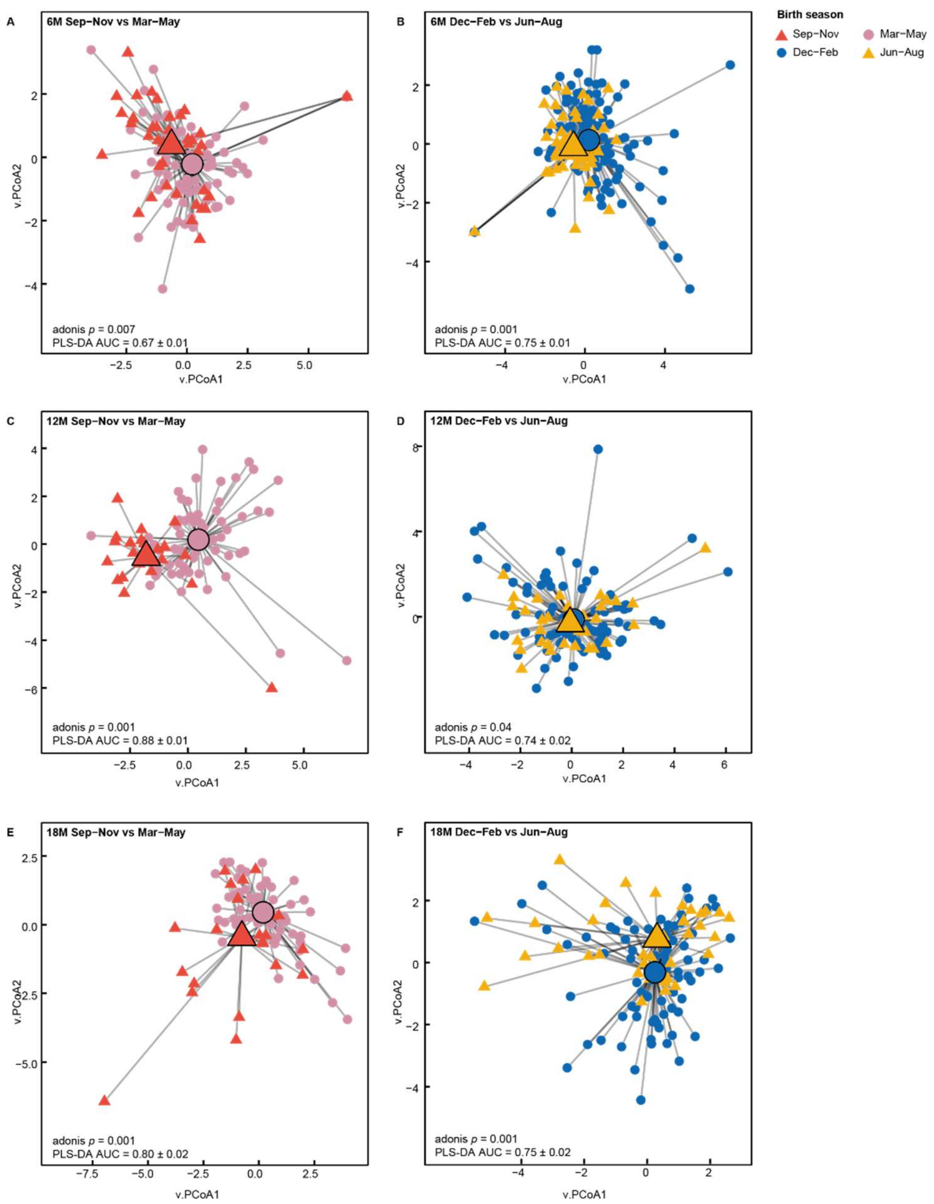

Participants clustered by birth season. PERMANOVA analysis using metabolites identified as following 12-month cosine distribution between those born in opposing seasonal windows at A-B) six (n = 278), C-D) 12 (n = 199), and E-F) 18-months (n = 199) of life. Color and shape demonstrate birth season (September-November, red, triangle; December-February, blue, circle; March-May, pink, circle; June-August, yellow, triangle). Group centroid outlined in black. Significance determined by *adonis*; AUC determined by cross-validated PLS-DA.

Fig.S6.

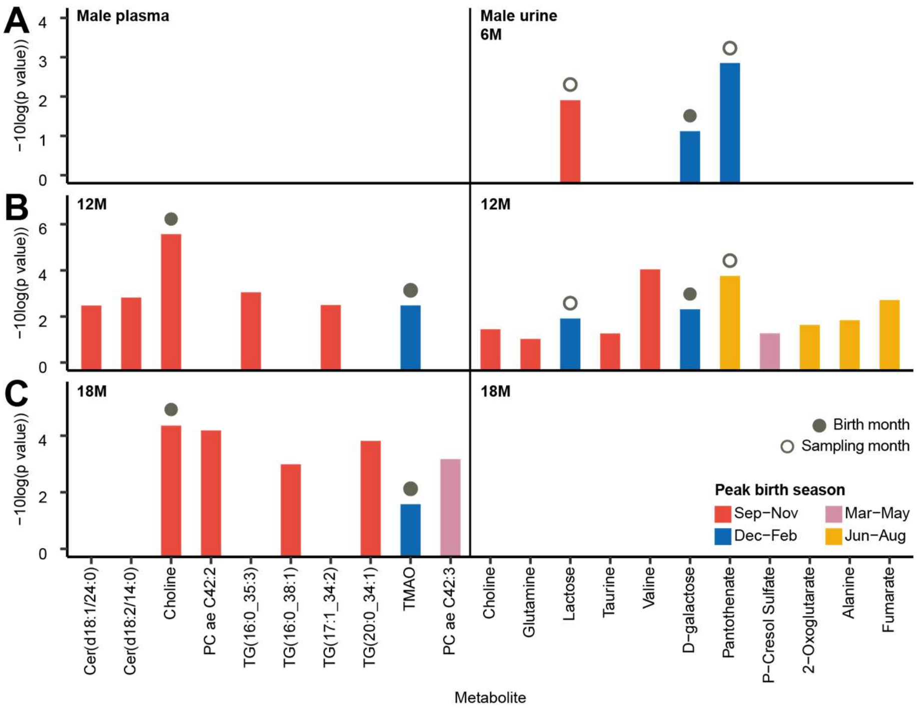

The male infant metabolome is more sensitive to environmental pressures. Plasma and urine metabolites that follow significant cosine distributions based upon birth month in males only at **A**) six months (n = 149), **B**) 12 months (n = 141), **C**) 18 months (n = 141; $p < 0.05$ & $q <$ 0.15 following Benjamini-Hochberg correction at ≥ one sampling point by extra-sums-of-squares F-test; exact p-values are provided in Supplementary Table 6) following adjustment for enrolment weight-for-age z-score, socioeconomic status, number of months of exclusive breastfeeding, and maternal factors (weight, height, age). Colored by birth season of greatest abundance (September-November, red; Dec-Feb, blue; March-May, pink; June-August, yellow). Solid circle represents birth-month-dependent metabolites (±1.5-month difference between birth month of peak abundance at two sampling points), unfilled circle indicates sampling-month-dependent (> four-month difference).

Fig.S7

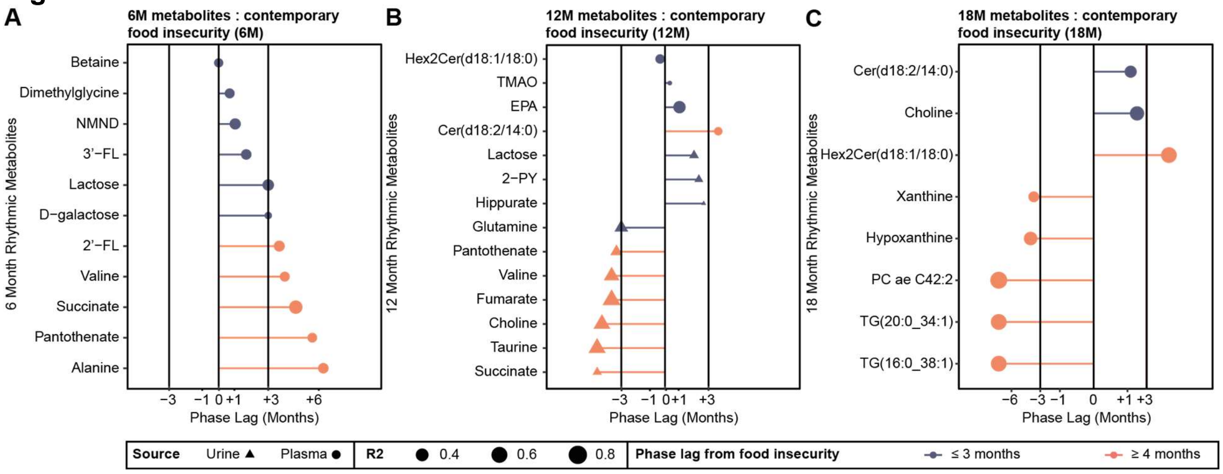

Cross-correlation between plasma and urinary metabolome at six, 12 and 18 months with contemporary food insecurity. A-C) Phase lag (months) from cross-correlation between contemporary food insecurity and plasma and urinary metabolites at six (n = 278 infants), 12 (n = 270 infants) and 18 months (n = 266 infants). Significant features have $q < 0.05$. Color indicates phase lag (> three months, purple; ≤ three months, pink); shape indicates source (urine, triangle; plasma, circle), and size R^2.

Fig.S8

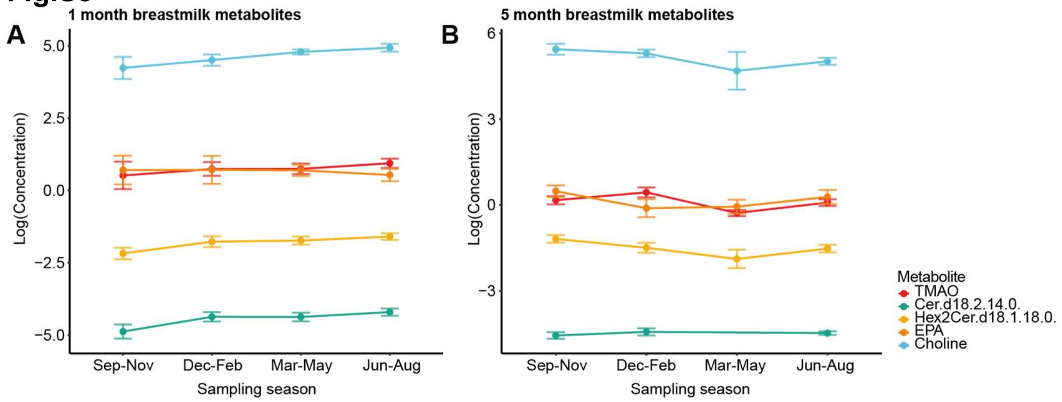

Birth-month-related plasma metabolites do not vary seasonally in the breastmilk. A-B) Breastmilk metabolites sampled at one- and five-months (1M n = 54; 5M n = 54) post-partum did not differ by birth season as assessed by Kruskal-Wallis test, two-sided $p > 0.05$. Colored by metabolite (trimethylamine *N*-oxide (TMAO), red; cer(d:18/2:14/0), green; hex2cer(d18/1:18/0), yellow; eicosapentaenoic acid (EPA), orange; choline, blue).

Fig.S9

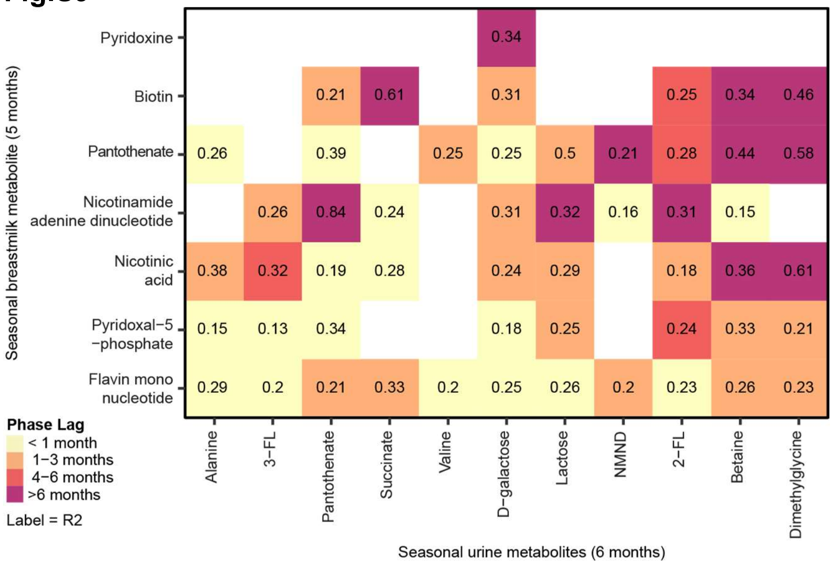

Transmission of seasonality from breastmilk to infant. Heat map of cross-correlation analysis between rhythmic five-month breastmilk vitamin-related metabolites and 6-month urinary metabolites (n = 278 infants). Significant features have $q < 0.05$. Color indicates phase difference between sampling months of peak abundance (> 6-month, purple; 4-6 month, red; 1-3 month, orange; < 1 month, yellow). Text indicates R^2 value.

Fig.S10

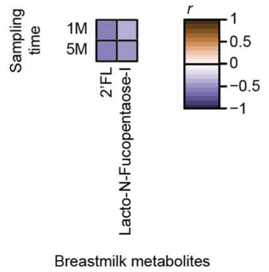

Correlation between seasonal breastmilk metabolites sampled at one- and five-months post-partum and cognitive outcomes at 18 months. Spearman's rank correlations (two-sided); significance determined by Benjamini-Hochberg corrected p-values ($q < 0.05$). $n = 54$ infants. Color indicates r value.

Fig.S11

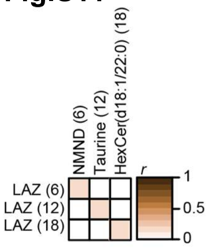

Correlation between systemic and urinary metabolites at 6, 12 and 18 months with contemporary and future growth. Spearman's rank correlations (two-sided) between urinary and plasma metabolites at six (*n* = 278 infants), 12 (*n* = 270 infants) and 18 months (*n* = 266 infants) months with Length-for-age-Z score (LAZ) at six, 12 and 18 months; significance determined by Benjamini-Hochberg corrected p-values ($q < 0.05$). Color indicates *r* value.

Fig.S12

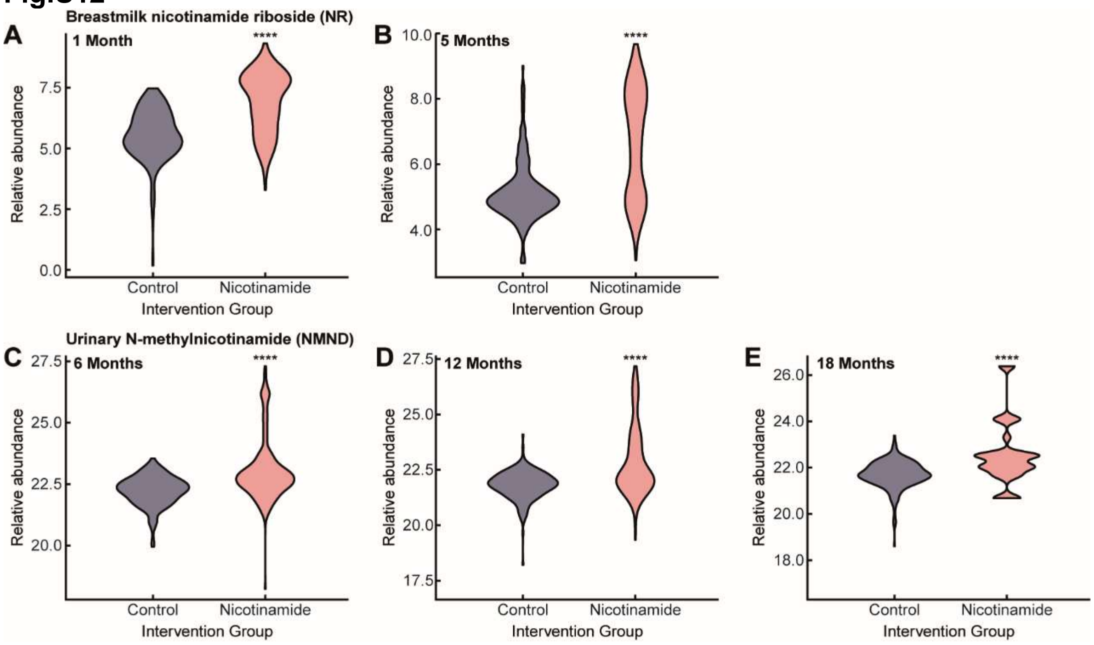

Nicotinamide supplementation increases breastmilk circulation and urinary excretion of nicotinamide-related metabolites. A-B) Violin plots showing relative abundance of breastmilk nicotinamide riboside at one- and five-months post-partum in control group (n = 292; purple) and those receiving nicotinamide supplementation (n = 295; pink). Data log10 transformed. Wilcoxon signed-rank test, two-sided. ****, $p < 0.0001$. **C-D)** Violin plots showing relative abundance of urinary N-methylnicotinamide (NMND) excretion in control group (purple) and those recieveing nicotianmide supplementation (pink) at 6 (control n = 278; nicotinamide supplemented n = 276), 12 (control n = 270; nicotinamide supplemented n = 267), and 18 months (control n = 266; nicotinamide supplemented n = 263) of life. Data log10 transformed. Wilcoxon signed-rank test, two-sided. ****, $p < 0.0001$.

Fig.S13

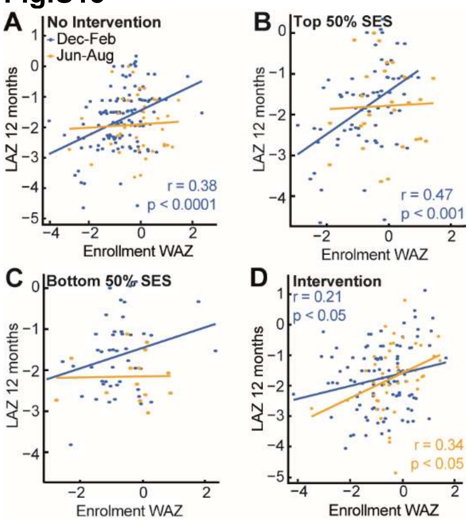

Correlation between enrolment WAZ and future LAZ depends on socioeconomic status (SES) and season of birth. Pearsons correlation (two-sided) between enrolment weight-for-age Z-score (WAZ) and 12-month length-for-age Z-score (LAZ) for **A)** non-supplemented infants (Dec-Feb born *n* = 116; Jun-Aug born *n* = 55); **B**) infants with the top 50% SES scores (December-February born *n* = 46; June-August born *n* = 18); **C)** infants with the bottom 50% SES scores (December-February born *n* = 70; June-August born *n* = 36); and **D**) nicotinamide supplemented infants (Dec-Feb born *n* = 114; Jun-Aug born *n* = 55). Color indicates season of birth (December-February, blue; June-August, yellow).

Fig.S14.

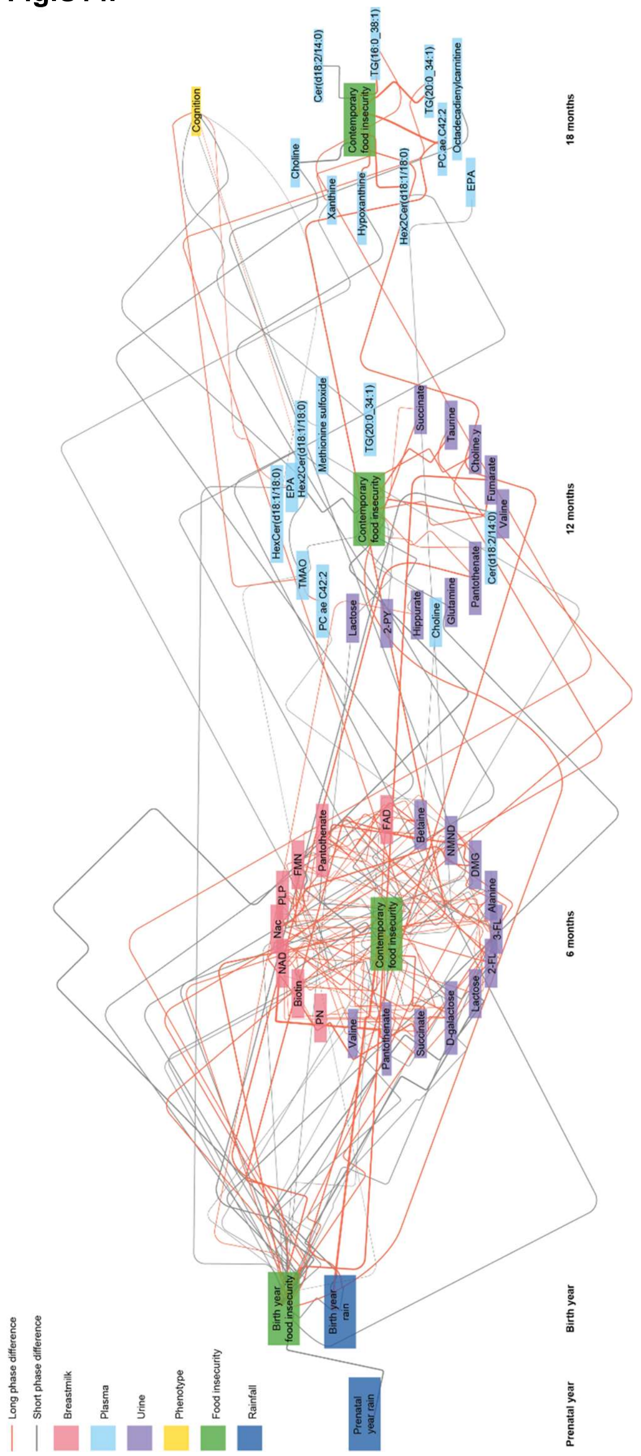

Interactome network linking rainfall and food insecurity with breastmilk, urinary and plasma metabolomes and phenotypic outcomes up to 18-months of life.
Lines demonstrate significiant ($q < 0.05$) cross correlations between features in the placebo group; colored by length of phase difference (time [months] between birth month associated with peak of feature; long ≥ four months (red); short = zero to three months(grey)). Features

colored by source (breastmilk, pink; plasma, light blue blue; urine, purple; phenotype, yellow; food insecurity, green; rainfall, dark blue). Line thickness is R^2.

Fig.S15

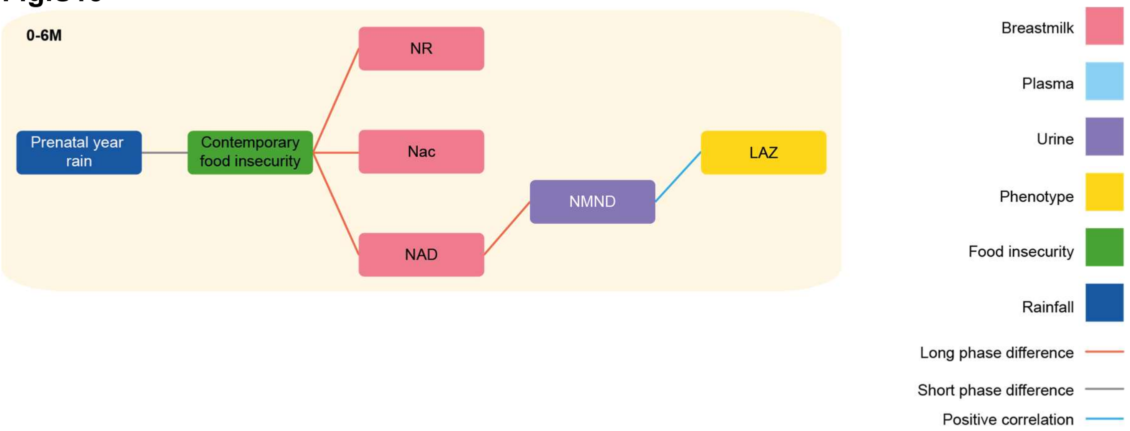

Interactome network linking rainfall and food insecurity with nicotinamide-related metabolites in breastmilk and urine and growth outcomes up to six-months of life. Lines demonstrate significiant (q < 0.05) correlations between features in the placebo group; colored by length of phase difference (time [months] between birth month associated with peak of feature; long ≥ four months (red); short = zero to three months (grey) for cross correlations and correlation direction for Spearmans correlations (positive, blue). Features colored by source (breastmilk, pink; plasma, light blue; urine, purple; phenotype, yellow; food insecurity, green; rainfall, dark blue).

Fig.S16.

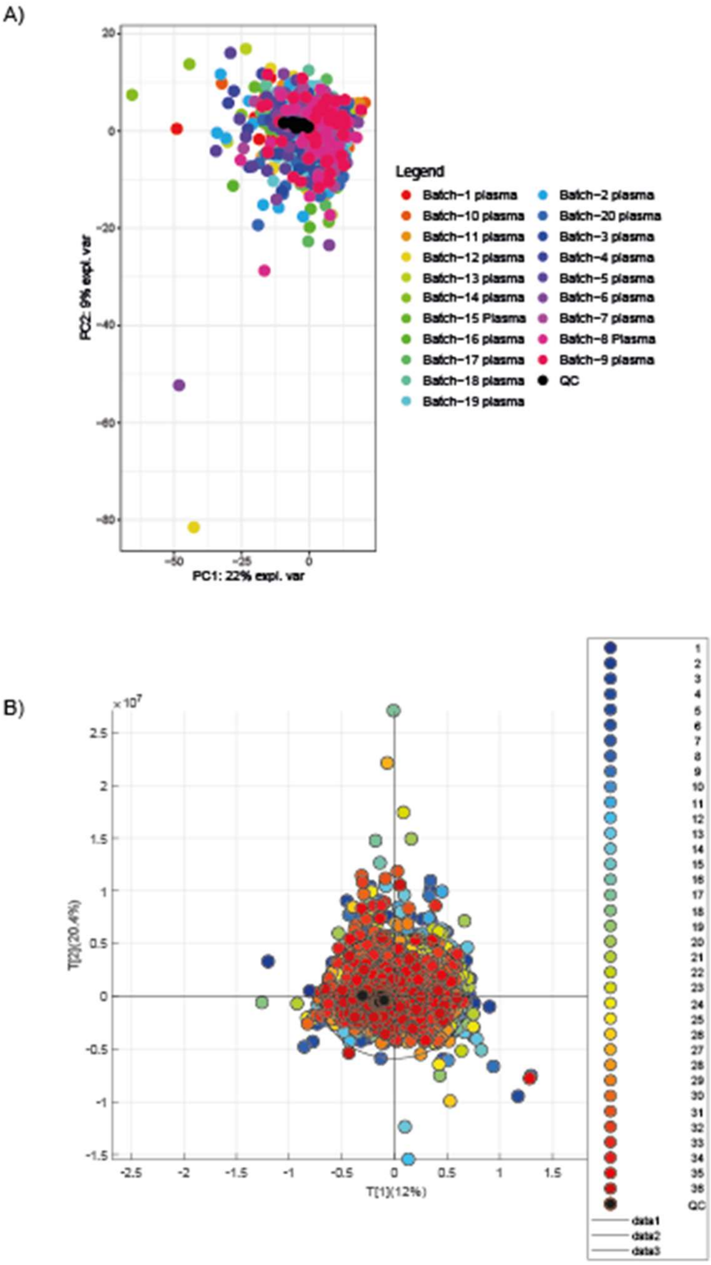

Quality control sample and batch distribution of plasma and urinary metabolomes
A) PCA scores plot of plasma metabolites colored by batch number. Median QC per batch colored black. B) PCA scores plot of urinary ^{1}H NMR spectra colored by batch number. Pooled biological QC per batch colored in black.

Supplementary Text

S.Text.1: Relationship between rainfall and food insecurity

Rainfall contributes to harvest stocks and ultimately food insecurity during the pre-harvest period of the following year, a pattern that has been explored in the literature (*54*). This was confirmed with cross-correlation analysis, identifying time-delayed relationships between monthly average rainfall and food insecurity. Significant correlations were noted between prenatal year (September 2016-17) monthly rainfall, and monthly reported food insecurity in the following year (R^2 = 0.54; p = 0.37 x 10-7; 365 days delay). Significant correlations were also observed in the next annual cycle between birth year (September 2017-18) rainfall and food insecurity the following year (R^2 = 0.48; p = 0.32 x 10-6; 375 days delay).Total rainfall in the year prior to enrolment (2016-17) was lower and shorter (493.1 mm) than the 8-year average (695.6 mm), and food insecurity was greater in the birth year compared to 12 months later (**Fig.3B**).

S.Text.2: Influence of food insecurity at birth on the infant metabolome

Food insecurity at birth cross-correlated with several seasonal metabolites at 6 and 12 months with a range of phase lags (**Fig.3C-D; Table.S8**). Short phase lags (≤90 days peak phase from peak food insecurity) demonstrated metabolites greatest in infants born during food scarcity. At 12 months this included lactose, methionine slufoxide, valine, glutamine and the phosphatidylcholine PC ae C42:2. At 6 months, short phase lags also suggested that infants born during peak food insecurity excreted the greatest amounts of breastmilk-related metabolites (2'-FL, 3'-FL, lactose, and D-galactose). However, as these were sampled 6 months after birth, mothers would have then been lactating during periods of food availability. In agreement with this observation, greater HMO production has been observed in Gambian mothers nursing during the dry season (*55*), a period of increased food availability.

S.Text.3: Influence of contemporary food insecurity on the infant metabolome

Cross-correlation analysis was additionally applied to explore the influence of contemporary food insecurity at the time of sampling on the seasonal metabolomic profiles (**Fig.S5A-C**; **Table .S9**). Several metabolites only aligned with contemporary food insecurity. For example, 2-PY in the urine at 12 months (phase lag = 70 days). This demonstrates the importance of current infant nutritional security dietary intake on these metabolites.

Due to the annual cycle of food availability, several metabolites cross-correlated with food insecurity during the birth year and with contemporary measures. Several of these were sampling time dependent determined by the ~6-month phase lag between sampling ages. Others only displayed seasonality at later points, therefore likely reflected current environmental pressures. Those with a short phase lag from food insecurity (≤90 days) were highest in infants sampled during food scarcity. This included urinary glutamine at 12 months; xanthine and hypoxanthine in plasma at 18 months. Conversely, metabolites with longer phase lags (~6 months) from food insecurity were increased during food availability. At 6 months, these included urinary valine, succinate, alanine, and pantothenate (vitamin B_5). Additionally, 12-month urine sampled during food availability had the greatest abundance of TCA cycle

intermediates (succinate and fumarate), and hippurate, a microbial-host co-metabolite associated polyphenol-containing foods (e.g., fruits and vegetables).

Statistical analysis plan:

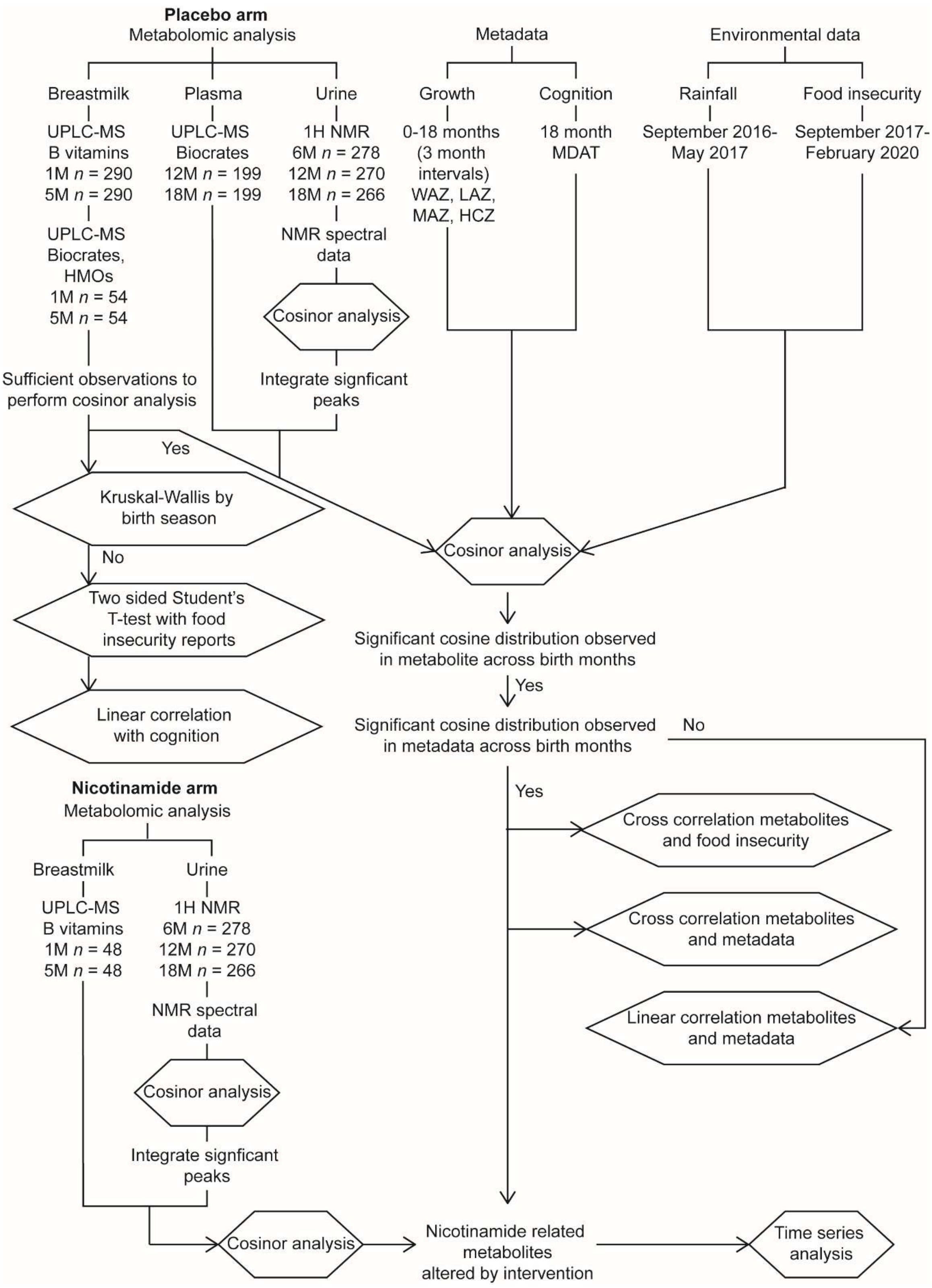

Supplement: Supplementary file 1 — Supplementary Information [file 41467_2025_66268_MOESM1_ESM.pdf]
