# Supplementary material for: Birth season shapes the infant metabolome and development in Tanzania: a secondary explorative analysis of the early life interventions for childhood growth and development in Tanzania (ELICIT) trial
Source: Nat Commun. 2025 Dec 13;16:11469. doi: 10.1038/s41467-025-66268-9 (PMC12749142; doi:10.1038/s41467-025-66268-9)
Supplement: Supplementary file 2 — Description of Additional Supplementary Files [file 41467_2025_66268_MOESM2_ESM.docx]

**Birth season shapes the infant metabolome and development in Tanzania: a secondary explorative analysis of The Early Life Interventions for Childhood growth and development in Tanzania (ELICIT) trial**

**Supplementary Data Legends:**

**Supplementary Data 1.** **Baseline characteristics of the cohort by intervention arm and birth season.**

Mean ± SD is shown for continuous variables and the number (percentage) for dichotomous variables unless otherwise stated.

**Supplementary Data 2: Plasma metabolites at 12 and 18 months follow significant cosine distribution across 12-month period based upon month of birth.**

Significant features *p* < 0.05 cosinor analysis by extra-sums-of-squares F-test. q values created by Benjamini Hochberg correction for multiple testing. Peak phase in days since September 1st 2017 (First enrolment day). Birth month dependent pattern for metabolites with ± 1.5-month difference between birth month of peak abundance at two sampling points. Sampling month dependent pattern for metabolites with > 4-month difference between birth month of peak abundance at two sampling points.

**Supplementary Data 3: Urinary metabolic features at 6, 12 and 18 months follow significant cosine distribution based upon month of birth.**

Integration of significant features *p* < 0.05 cosinor analysis by extra-sums-of-squares F-test. *q* values created by Benjamini Hochberg correction for multiple testing. Peak phase in days since September 1st 2017 (First enrolment day). Birth month dependent pattern for metabolites with ±1.5-month difference between birth month of peak abundance at two sampling points. Sampling month dependent pattern for metabolites with > 4-month difference between birth month of peak abundance at two sampling points.

**Supplementary Data 4: VIP scores of PLS-DA models classifying into birth season.**

VIP scores for PLS-DA models classifying participants into opposing birth seasons utilizing metabolites identified as following a 12-month cosine distribution by birth month at six, 12 and 18 months of life.

**Supplementary Data 5: Metabolite abundance and concentration by birth season.**

Mean ± SD grouped by birth season. Concentration (μM) displayed for Plasma metabolites and lipids as assessed by MS; relative abundance displayed for urinary metabolites from 1H NMR. Features have *p* < 0.05 & *q* < 0.15 in cosinor analysis.

**Supplementary Data 6: Sex-differences in the seasonal patterns of plasma and urinary metabolites at 6-18 months.**

Significant features *p* < 0.05 cosinor analysis by extra-sums-of-squares F-test. *q* values created by Benjamini Hochberg correction for multiple testing. Peak phase in days since September 1st 2017 (First enrolment day). ͨ Amplitude of concentration (uM) for plasma metabolites, and relative abundance for urinary metabolites.

**Supplementary Data 7: Cross correlation between 18-month MDAT cognitive function scores and 12-month plasma metabolites.**

Significant features from cross correlation analysis *q* < 0.05 after Benjamini-Hochberg correction for multiple testing.

**Supplementary Data 8: Cross correlation between food insecurity at birth and urinary and plasma metabolites at 6, 12 and 18 months that exhibit seasonality.**

Significant features from cross correlation analysis *q* < 0.05 after Benjamini-Hochberg correction for multiple testing. Birth year, September 2017-August 2018.

**Supplementary Data 9: Cross correlation between contemporary food insecurity and urinary and plasma metabolites that exhibit seasonality at 6, 12 and 18 months.**

Significant features from cross correlation analysis *q* < 0.05 after Benjamini-Hochberg correction for multiple testing. Contemporary food insecurity, 6 months February 2018-January 2019; 12 months September 2018-August 2019; 18 months February 2019-January 2020.

**Supplementary Data 10: Seasonally varying breastmilk metabolite abundance by birth season.**

Mean concentration (μM) ± SD grouped by birth season. B-vitamin-related metabolites were measured in all participants and selected features have *p* < 0.05 & *q* < 0.15 in cosinor analysis. Human milk oligosaccharides and metabolites were measured in a subset (*n* = 54) and selected features have *q* < 0.05 in Kruskal-Wallis and post-hoc testing comparing binned birth seasons.

**Supplementary Data 11: Breastmilk metabolites at 1- and 5-months post-partum follow significant cosine distribution based upon month of sampling.**

Significant features *p* < 0.05 cosinor analysis by extra-sums-of-squares F-test. *q* values created by Benjamini Hochberg correction for multiple testing. Peak phase in days since sampling month October 1st 2017 (1 Month), January 1st 2018 (5 Months).

**Supplementary Data 12: Breastmilk metabolites at 1- and 5-months post-partum that vary by season of sampling in ANOVA or Kruskal Wallis and post hoc testing.**

Significant features *q* < 0.15 after Benjamini-Hochberg correction for multiple testing.

**Supplementary Data 13: Cross-correlation analysis between rhythmic vitamin-related breast milk metabolites sampled at 1 and 5 months and food insecurity at the time of sampling.**

Significant features from cross correlation analysis *q* < 0.05 after Benjamini-Hochberg correction for multiple testing. Food insecurity, 1 month October 2017-September 2018; 5 months January 2018-December 2018.

**Supplementary Data 14: Breastmilk metabolites sampled at one-month post-partum that significantly differ between mothers who report food insecurity from those who did not at the time of sampling.**

Wilcox test (two-sided). Significant features *q* < 0.05

**Supplementary Data 15: Cross-correlation analysis between rhythmic vitamin-related breast milk metabolites sampled at 5 months and seasonal 6-month urinary metabolites.**

Significant features from cross correlation analysis *q* < 0.05 after Benjamini-Hochberg correction for multiple testing.

**Supplementary Data 16: Correlation between season-dependent breastmilk metabolites (sampled at one and five months) and total MDAT/language scores at 18 months.**

Spearman's rank correlations (two-sided); significance determined by Benjamini-Hochberg corrected p-values (*q* < 0.05).

**Supplementary Data 17: Growth measures do not follow significant cosine distribution across 12-month period based upon month of birth.**

Significant features *p* < 0.05 cosinor analysis by extra-sums-of-squares F-test. *q* values created by Benjamini Hochberg correction for multiple testing. Peak phase in days since September 1st 2017 (First enrolment day).

**Supplementary Data 18: Correlation between plasma and urinary metabolites at 6, 12 and 18 months with contemporary and future growth.**

Spearman's rank correlations (two-sided); significance determined by Benjamini-Hochberg corrected p-values (*q* < 0.05).

**Supplementary Data 19: Birth season dependent nicotinamide response for those with upper tertile enrolment weights.**

Results of the interaction effect of birth season and nicotinamide supplementation on change in LAZ from 0-18 months in a two-way ANOVA performed on infants with top tertile enrolment WAZ (≤ -0.13), followed by post hoc Tukey test. Significant features *q* < 0.05 for Benjamini Hochberg corrected p-values.
