# Supplementary material for: Birth season shapes the infant metabolome and development in Tanzania: a secondary explorative analysis of the early life interventions for childhood growth and development in Tanzania (ELICIT) trial
Source: Nat Commun. 2025 Dec 13;16:11469. doi: 10.1038/s41467-025-66268-9 (PMC12749142; doi:10.1038/s41467-025-66268-9)
Supplement: Supplementary file 4 — Reporting Summary [file 41467_2025_66268_MOESM4_ESM.pdf]

nature portfolio

Corresponding author(s): Jonathan Swann

Last updated by author(s): Oct 8, 2025

Reporting Summary

Nature Portfolio wishes to improve the reproducibility of the work that we publish. This form provides structure for consistency and transparency in reporting. For further information on Nature Portfolio policies, see our Editorial Policies and the Editorial Policy Checklist.

Statistics

For all statistical analyses, confirm that the following items are present in the figure legend, table legend, main text, or Methods section.

n/a	Confirmed	
☐	☒	The exact sample size (*n*) for each experimental group/condition, given as a discrete number and unit of measurement
☐	☒	A statement on whether measurements were taken from distinct samples or whether the same sample was measured repeatedly
☐	☒	The statistical test(s) used AND whether they are one- or two-sided<br>*Only common tests should be described solely by name; describe more complex techniques in the Methods section.*
☐	☒	A description of all covariates tested
☐	☒	A description of any assumptions or corrections, such as tests of normality and adjustment for multiple comparisons
☐	☒	A full description of the statistical parameters including central tendency (e.g. means) or other basic estimates (e.g. regression coefficient) AND variation (e.g. standard deviation) or associated estimates of uncertainty (e.g. confidence intervals)
☐	☒	For null hypothesis testing, the test statistic (e.g. *F*, *t*, *r*) with confidence intervals, effect sizes, degrees of freedom and *P* value noted<br>*Give P values as exact values whenever suitable.*
☒	☐	For Bayesian analysis, information on the choice of priors and Markov chain Monte Carlo settings
☒	☐	For hierarchical and complex designs, identification of the appropriate level for tests and full reporting of outcomes
☐	☒	Estimates of effect sizes (e.g. Cohen's *d*, Pearson's *r*), indicating how they were calculated

Our web collection on statistics for biologists contains articles on many of the points above.

Software and code

Policy information about availability of computer code

Data collection	Topspin_3.2 was used to collected NMR spectral data, which was processed using MATLAB_R2022b using the Imperial Metabolic Profiling and Chemometrics Toolbox (https://github.com/csmsoftware/IMPaCTS). Biocrates MetIDQ software (version Oxygen-DB110-302305) was used to process the mass spectrometry data.
Data analysis	MATLAB_R2022b (Statistics and Machine Learning Toolbox; Optimization Toolbox; Bio informatics Toolbox, Signal Processing Toolbox), and R_4.2.1 (stats (4.2.1), vegan (2.6.10), mixOmics (3.21), santaR (1.2.3), or dplyr (1.1.10) packages) were used to analyze all the data presented.

For manuscripts utilizing custom algorithms or software that are central to the research but not yet described in published literature, software must be made available to editors and reviewers. We strongly encourage code deposition in a community repository (e.g. GitHub). See the Nature Portfolio guidelines for submitting code & software for further information.

Data

Policy information about availability of data

All manuscripts must include a data availability statement. This statement should provide the following information, where applicable:

- Accession codes, unique identifiers, or web links for publicly available datasets
- A description of any restrictions on data availability
- For clinical datasets or third party data, please ensure that the statement adheres to our policy

Metabolomic, cognition and environmental data are provided in the Source Data file.

Research involving human participants, their data, or biological material

Policy information about studies with human participants or human data. See also policy information about sex, gender (identity/presentation), and sexual orientation and race, ethnicity and racism.

Reporting on sex and gender	Both sex combined and segregated analyses were performed and reported in the manuscript. Baseline characteristics including sex have been detailed in supplementary table 1. Sex was determined by self-report.
Reporting on race, ethnicity, or other socially relevant groupings	Socioeconomic status was assessed as quartiles of Water and sanitation, Assets, Maternal education, and household Income (WAMI) scores (1-4) at the first study visit. This was performed by a study team member and answered by a parent/guardian of the participant. This score summarized the variables: score of sanitation, drinking water source, household assets (e.g. family possession of mattress, chair, table, TV, refrigerator, bank account, kitchen, <2 people per room), income in US dollars. Improved drinking water source included a household connection, public standpipe, borehole, protected dug well, protected spring, or rainwater collection. Improved sanitation facilities were connected to a public sewer or septic system, a pour-flush latrine, or a ventilated improved pit latrine. Treated water referred to water that was boiled, filtered, or treated with bleach. Analysis was performed comparing the top 50% against the bottom 50% of WAMI scores, or it was included as a covariate.
Population characteristics	Participants were aged between 0 and 18 months.
Recruitment	Mothers were identified at Haydom Lutheran Hospital, Haydom, Tanzania by community health workers if they were either pregnant or had recently delivered within the recruitment area. These pregnant women and mothers were then approached at their homes by field team members to inform them about the study and assess interest. Inclusion criteria were maternal age ≥18 years, child age ≤14 days, and the family's stated intent to reside within a 25-km radius of Haydom Lutheran Hospital for the duration of the study. Exclusion criteria were multiple gestation, significant birth defect or neonatal illness, infant enrollment weight <1,500 g, and lack of intent to breastfeed.
Ethics oversight	The study protocol was approved by the National Institute for Medical Research (NIMR) of Tanzania and the Tanzanian Food and Drug Administration (TFDA) and the Institutional Review Board at the University of Virginia. Study oversight was provided by FHI360 (North Carolina, United States of America). This study was registered in ClinicalTrials.gov (NCT03268902) prior to the start of enrollment. Mothers gave written informed consent to participate either during pregnancy or at the time of enrollment.

Note that full information on the approval of the study protocol must also be provided in the manuscript.

Field-specific reporting

Please select the one below that is the best fit for your research. If you are not sure, read the appropriate sections before making your selection.

☒ Life sciences ☐ Behavioural & social sciences ☐ Ecological, evolutionary & environmental sciences

For a reference copy of the document with all sections, see nature.com/documents/nr-reporting-summary-flat.pdf

Life sciences study design

All studies must disclose on these points even when the disclosure is negative.

Sample size	Sample sizes were determined to provide sufficient power to test the primary study outcomes of difference between intervention groups in LAZ at 18 months. The sample size was calculated to provide sufficient power for testing the main effects using separate t-tests for each of the two interventions in this 2×2 factorial design, using a SD of 1.03 HAZ at 18 months. If each main effect were tested at the 5% levels, as is customary in the analysis of factorial studies with 270 participants per group, there was 80% power for a difference in LAZ of 0.176. This is within the range of improvement of an increase of 0.16 seen in a prior study after 12 months of micronutrient supplementation though in Haydom the growth and deficiencies are more severe than most areas, thus the potential for observing a greater effect size. Within our recruitment radius >2507 births occur yearly. We recruited over a 12-month window. In our extensive research experience at the Haydom MAL-ED site, we observed 0% refusal to participate and a high 81% retention rate by 4 years of follow-up during MAL-ED (and, thus, a study of shorter duration may have improved follow-up). Based on this, we anticipated a high rate of recruitment (>90%) and 18-month retention (>90%). Within this group size, sufficient infants were recruited to be born distributed across the year from September 2017-August 2018. As this manuscript was a secondary, exploratory analysis, the sample size could only include those determined for the main RCT.
Data exclusions	Metabolic variables with greater than 80% missing values (i.e., those below the limit of detection) were excluded.
Replication	This was a human study (n = 278 placebo; n = 276 intervention) therefore individual level replication was not possible. Technical reproducibility was ensured using pooled, or manufacturer provided, quality control samples throughout metabolomic analyses to monitor instrument performance and data stability over time. Statistical robustness was evaluated through cross-validation and sensitivity analyses.
Randomization	Individuals were allocated to one of 4 treatment groups (1:1:1:1) in a 2 × 2 factorial manner, such that participants received either nicotinamide plus placebo, placebo plus antimicrobials, both interventions, or both placebo. In this manuscript, only infants from the nicotinamide/placebo and placebo/placebo arms of the study were considered. Individuals were randomly allocated to treatment arms using permuted blocks with a block size of 8 and a reproducible seed by study author JPM. Lists of the allocation blocks were stored at the research center and provided to the field teams to randomize participants at enrollment.
Blinding	All investigators and participants remained blinded to treatment assignment until after study completion and the analysis of the pre-specified

Blinding

primary outcome. Allocation for each study intervention was concealed by the manufacturer, who provided both the active treatment and matching placebo, with the allocation code held subsequently held in a sealed opaque envelope by a non-study investigator. Investigators were unblinded during metabolomic data collection, however samples were randomized across the analytical runs.

Reporting for specific materials, systems and methods

We require information from authors about some types of materials, experimental systems and methods used in many studies. Here, indicate whether each material, system or method listed is relevant to your study. If you are not sure if a list item applies to your research, read the appropriate section before selecting a response.

Materials & experimental systems

n/a	Involved in the study	
☒	☐	Antibodies
☒	☐	Eukaryotic cell lines
☒	☐	Palaeontology and archaeology
☒	☐	Animals and other organisms
☐	☒	Clinical data
☒	☐	Dual use research of concern
☒	☐	Plants

Methods

n/a	Involved in the study	
☒	☐	ChIP-seq
☒	☐	Flow cytometry
☒	☐	MRI-based neuroimaging

Clinical data

Policy information about clinical studies

All manuscripts should comply with the ICMJE guidelines for publication of clinical research and a completed CONSORT checklist must be included with all submissions.

Clinical trial registration

NCT03268902

Study protocol

DOI: 10.1136/bmjopen-2018-021817

Data collection

Monthly home visits were performed by study team personnel from 1 to 18 months of life (± 7 days). At each monthly visit mothers were asked about breastfeeding status, childhood illness and treatment, intervention compliance, and household food insecurity. . At each 3-month visit, anthropometric data was obtained for the participants by trained field team members using a standard operating protocol. Length was assessed by two field members using a measuring board. This involved the infant lying flat on the board, with two perpendicular boards (one placed above the head and one below the feet) dictating the measurement. This was taken to the nearest millimeter, with an average of two measurements taken. If these were not within 2mm of each other, a third was taken and the average of the two closest utilized for analysis. Participant weight was measured in kilograms by digital scales to the nearest 10g. Head circumference was measured using a non-distensible measuring tape in centimeters to the nearest millimeter. This tape was placed above the ears and eyebrows, around the largest part of the back of the infant's head. Mid-upper arm circumference was measured using the same non-distensible measuring tape placed around the mid-point of the infant's upper arm.

Blood samples were collected at 12 and 18 months of life. A butterfly needle and syringe were used to draw the blood sample by an experienced field team member. It was then transferred into micro-containers for transportation to the laboratory center. Here, the whole blood was processed into plasma and serum samples and stored at -80°C, before being shipped to the United Kingdom for metabolomic analysis.

Urine samples were collected at 6, 12, and 18 months. When the study visit began, the infant's genitalia was covered with a bag to facilitate collection throughout the visit. During this time, the infant was kept sufficiently hydrated, ideally though breastfeeding from the mother. At the end of the study visit, the urine was then collected into a sterile container for transportation on ice to the laboratory center where it was stored at -80°C, before being shipped to the United Kingdom for metabolomic analysis.

Breastmilk was collected at 1- and 5-months post-partum. Mothers manually expressed up to 40 ml of breastmilk from one breast into foil-covered sterile containers before transportation to the laboratory center within eight hours on ice. Here, it was stored at -80°C and shipped to the United States of America for analysis.

Rainfall data was collected from the weather station in Haydom including historic rainfall from July 2010-July 2018.

Developmental assessments were performed at 18 months, including the Malawi Developmental Assessment Tool (MDAT). This test has been designed and validated to test gross and fine motor, language, and social development domains in a culturally relevant manner for rural or lower-resource settings in sub-Saharan areas with good reliability (94-100%). For Haydom specifically, the MDAT has been specifically adapted, piloted, and validated by the MAL-ED study team. The assessment was delivered to participants in Swahili or Iraqw (the first language of most of the participants) by three trained members of the cognitive field team. To ensure accuracy in translation, the assessment was then translated into Swahili and back translated into English by a second independent interpreter. For each task, individuals were scored either 1 (pass) or 0 (not pass), where they continued completed tasks in each subtest until they completed six tasks consecutively with a score of 0. To monitor consistency, all assessments were video recorded with 10-20% of these were watched back each week by trained cognitive team study personnel.

Outcomes

This manuscript contains a secondary analysis of data obtained from the ELICIT randomized control trial. The primary and secondary

Outcomes	outcomes of this study have been documented in the study design (doi:10.1136/bmjopen-2018-021817) and outcomes publications (doi:10.1371/journal.pmed.1003617).
